# Is it Genuine or Pseudo-Forgiveness? Offenders’ Appraisals of Victims’ Expressed Forgiveness as a Function of Engagement in Co-Reflection

**DOI:** 10.5334/irsp.887

**Published:** 2024-08-19

**Authors:** Blake Quinney, Michael Wenzel, Michael Thai, Tyler Okimoto, Lydia Woodyatt

**Affiliations:** 1 Flinders University, AU; 2 University of Queensland, AU

**Keywords:** forgiveness, value consensus, conflict resolution, co-rumination

## Abstract

After interpersonal wrongdoing, a victim may express forgiveness with or without having truly experienced a transformation to more positive sentiments toward the offender. As those forgiving sentiments are internal states, offenders do not know, and would need to make inferences, whether the forgiveness is genuine or pseudo-forgiveness. Two studies, an experiment using vignettes (*N* = 308) and a correlational study using a recalled wrongdoing (*N* = 179), provided evidence that, to the extent that the forgiveness was preceded by a reflective dialogue with the victim (i.e., co-reflection), offenders perceived the victim to believe in a shared value consensus and, mediated by it, appraised the forgiveness as more genuine. These findings highlight the dyadic nature of the moral repair process: the victim’s forgiveness gains meaning through the offender’s appraisal. If a victim wishes to communicate genuine forgiveness, then engaging with the offender in co-reflection may facilitate such meaning.

Interpersonal transgressions are violations of rules, norms, or moral codes, whereby one person harms, insults, or betrays another person. While transgressions are certainly psychologically damaging to victims, offenders who commit the transgression can fear social exclusion, rejection and the loss of respect or acceptance by the victim and the wider community (Woodyatt et al. 2022). To address threats arising from transgressions, victims and offenders may engage in *moral repair*, the process of rebuilding trust and cultivating hope for a future moral relationship (Wenzel et al. 2021). Offenders who engage in moral repair processes should accept the onus of relationship repair given they are responsible for the hurt (Andrews 2000), and they should not expect to receive forgiveness in return. Yet, victims may choose to express forgiveness towards the offender (Baumeister et al. 1998). However, being told ‘I forgive you’ can have multiple potential meanings for the person receiving the forgiveness. Although victims may express forgiveness to communicate a genuine wish for their relationship to be restored (Takada & Ohbuchi 2013), their expressed forgiveness could also reflect *pseudo-forgiveness* when they still hold unforgiving sentiments (Zechmeister & Romero 2002), or a wish to avoid the issue or minimise the impact of the transgression. The different meanings that expressed forgiveness could hold present a conundrum: if victims wanted to express *genuine* forgiveness, they could not be sure that offenders would understand it as such. Because forgiveness is an internal state, offenders do not have direct knowledge of whether victims truly mean it when they forgive. How do offenders then come to believe that the forgiveness is genuine and not pseudo-forgiveness?

Little research has looked at the expression of forgiveness from the offender’s perspective and how they appraise victim’s forgiveness. Research is often focussed on victims, investigating questions such as how forgiveness can improve the well-being of victims (Enright & Fitzgibbons 2015); offenders’ perceptions of the victims’ forgiveness may not matter for its effects on victims’ personal well-being. However, forgiveness can also help restore damaged relationships and maintain valued relationships (McCauley et al. 2022), and victims can communicate forgiveness out of concern for their relationship with the offender (Takada & Ohbuchi 2013) or even for the well-being of the offender (Karremans & Van Lange 2004). When motivated by concern for the offender or the relationship, victims may want their forgiveness to be seen as genuine, as being true to their feelings, communicating their transformed sentiments toward the offender. However, offenders cannot directly observe the sentiment behind the expression of forgiveness because these are internal processes. Thus, offenders would not know whether the forgiveness is genuine or pseudo-forgiveness and must make attributions (Gollwitzer & Okimoto 2021).

While forgiving sentiments are internal, the processes through which victims arrive at them may be observable or shared, allowing offenders to use these to make appraisals and inferences about whether the forgiveness expressed is genuine or pseudo-forgiveness. We reason that if offenders see the forgiveness as an outcome of co-engagement or joint discussions on the issue with the victim, then they will be more likely to perceive genuine forgiveness (and less likely to perceive pseudo-forgiveness). We theorise that co-engagement allows offenders to witness how victims arrive at the forgiveness and appraise the mental work and transformation that led them to it. Hence, it is the processes of social interaction or co-engagement that should give offenders insight into whether the expression of forgiveness is genuine or not.

## The Perceived Meaning of Expressed Forgiveness After Wrongdoing

*Forgiveness* is typically defined as a transformation from negativity and ill-will towards the offender to positivity and goodwill (Worthington 2019). Victims may explicitly communicate their forgiveness to the offender, or implicitly, by showing a change in attitude or disposition towards the offender (Wohl et al. 2006). Just as there are differences in how forgiveness is communicated, there are also different possibilities for what forgiveness means.

One possibility is *genuine forgiveness*, when the expression of forgiveness is paired with an internal forgiving sentiment (Baumeister et al. 1998). Typically, victims offer genuine forgiveness when motivated to restore or maintain their relationship with the offender (Takada & Ohbuchi 2013). Genuine forgiveness perhaps best captures the type of forgiveness that is considered a ‘gift’ from the victim to the offender (Enright 2001; Worthington 2001).

However, victims can express forgiveness without genuine attitude change or intent to forgive, termed *pseudo-forgiveness* (Enright 2001) or *hollow forgiveness* (Baumeister et al. 1998). One possibility is that victims say they forgive, but feel *unforgiveness*, an internal state of unforgiving emotions, cognitions, and/or an unfavourable perception of the offender (Stackhouse et al. 2018). Researchers often study unforgiveness and grudges when victims explicitly withhold forgiveness (van Monsjou et al. 2022), but victims can express forgiveness yet still feel anger or varying degrees of unforgiveness (Zechmeister & Romero 2002). Importantly, at times, offenders can detect unforgiveness when victims say they forgive (Strelan et al. 2017), or even appraise the expression of forgiveness as a retaliatory act borne of unforgiveness (e.g., to demean the offender or put them in debt; Worthington & Wade 1999).

Another possibility is that victims may express forgiveness to brush the incident aside without acknowledging the hurt, reasonably apportioning responsibility, or making sense of the incident. This may reflect *avoidance*, where the victim may try to disengage completely by attempting to ignore or forget the incident. It could also reflect *minimisation*, where the victim may try to downplay the impact of the incident or minimise its significance which forgiveness theorists view as conceptually distinct from forgiveness (Worthington 2019). The function of these acts of forgiveness may be a form of self-persuasion that the wrongdoing did not bother them, perhaps as an effort to remove the threat imposed by the wrongdoing (Gabriels & Strelan 2018). Offenders can underestimate the harm they caused victims and so offenders could view victims’ avoidance and minimisation as appropriate responses in some circumstances (Baumeister et al. 1990). Nevertheless, these expressions are still pseudo-forgiveness as there is no genuine sentiment behind the forgiveness.

We outline different possibilities for victims’ forgiveness, but we do not suggest victims who express pseudo-forgiveness are doing so because of immoral character or maladaptive behaviour (see also Stackhouse et al. 2018). There are circumstances where victims may not feel genuinely forgiving but nonetheless may feel compelled or expected to express forgiveness (Fehr & Gelfand 2012) or would face potential/real costs to withholding forgiveness from offenders (Rapske et al. 2010). To be clear, victims have the right to express forgiveness without full conviction and move on. But in cases where victims desire reconciliation, an understanding of what leads offenders to perceive genuine forgiveness is critical for how offenders view the standing of the relationship. Moreover, the different ways that offenders can interpret victims’ forgiveness can have consequences for whether they commit to reconciliation. For example, Mooney et al. (2016) asked participants to imagine committing a wrongdoing and then being forgiven by the victim, but for different reasons. Participants who were informed that the forgiveness was for selfish reasons (vs. benevolent forgiveness) were less willing to reconcile with the victim.

In real instances of expressed forgiveness, offenders cannot directly observe the sentiment behind forgiveness and so when victims forgive, offenders only receive the message of forgiveness, and how the forgiveness was communicated (Gollwitzer & Okimoto 2021). Given the different meanings that forgiveness could entail, offenders must appraise the expressed forgiveness and decide whether they believe in its veracity. Yet, how do offenders appraise forgiveness, and on what basis do they decide whether forgiveness is genuine or not? We theorise that offenders base their forgiveness evaluations on their interactions with victims (Kelley et al. 2018). Accordingly, the social processes shared between victim and offender preceding the forgiveness may shape the appraisals that offenders make about victims’ forgiveness. We therefore examine how offenders appraise the genuineness of expressed forgiveness as a function of victim-offender engagement.

## Appraisals That Shape the Meaning of Victims’ Expressed Forgiveness

An offender may appraise a victim’s forgiveness by considering whether they believe the victim has put mental resources into *processing* the incident. Perceiving that the victim has spent time working through the incident may suggest to the offender that, at the very least, the victim has engaged with the incident (i.e., the victim is not avoiding), and has recognised that the transgression had an impact (i.e., the victim is not minimising). However, offenders may view processing as somewhat of a ‘black box’ because it simply implies that the victim has cognitively engaged with the wrongdoing, but not *how* they have thought about it, or what they now think about the incident. Thus, perceived processing may be associated with less avoidance and minimisation, but not necessarily with greater genuine forgiveness or lower unforgiveness.

Another key appraisal for genuine forgiveness may be the extent to which the offender believes the two parties are on the same page about what is important and underpins their shared relationship – termed *value consensus*. Wrongdoing undermines shared values (Okimoto & Wenzel 2008), and regaining consensus on these violated values is critical when reconciliation is the aim, because this would establish a regained trust that the future relationship is grounded in. Past work demonstrates that when a victim communicates forgiveness to an offender (following an interpersonal transgression) it signals to the offender that the victim feels that they are back on the same page (i.e., *meta-perceived value consensus*), which, in turn, influences the offender to believe in such consensus themselves (i.e., ‘we are on the same page’), facilitating their own genuine self-forgiveness (Wenzel et al. 2021; 2023). Beyond promoting their own genuine self-forgiveness, it is possible that meta-perceived value consensus also may lead the offender to believe in the genuineness of the expressed forgiveness and that the victim has let go of their resentment or unforgiveness because the offender may intuit ‘You are forgiving me because you know that I know what is really at stake here, and I am committed to those shared values.’

In sum, offenders may not necessarily interpret victims’ expressed forgiveness as genuine, but certain appraisals (i.e., processing, and meta-perceived value consensus) may imply the forgiveness is genuine and not pseudo-forgiveness. But on what basis do offenders make these appraisals? We test the proposition that the type of engagement that follows wrongdoing (i.e., how the wrongdoing is addressed) and precedes the expression of forgiveness shapes the perceived meaning of victims’ expressed forgiveness.

## Engagement: Co-Reflection as a Pathway to Perceiving Forgiveness as Genuine

Engagement can be divided into individual and dyadic forms, with the key differentiation being whether engagement is solitary or interpersonal. Individual engagement may occur as *individual reflection*, which involves contemplation of the issue and cognitive/emotional processing, by oneself (Woodyatt et al. 2022). Certain actions by the victim, such as taking time or space to be by themselves (or away from the offender), may indicate to the offender that the victim is engaging in individual reflection. Engagement can also be dyadic (Wenzel et al. 2022), and the evidence suggests that *co-reflection*, the collaborative working through of the incident with the other party, is an effective dyadic approach to resolving interpersonal conflict (Thai et al. 2023).

We propose that offenders’ appraisals of forgiveness are partly determined by the extent to which victim and offender have engaged with the wrongdoing and with each other. For example, both individual reflection and co-reflection imply that the victim has spent time *processing* the incident and so may inform the offender that the victim is not forgiving as a form of avoidance or minimisation. However, unlike individual reflection, co-reflection is interpersonal and interactive. This may have additional implications for how offenders view victims’ forgiveness. In particular, the reestablishment of a shared consensus on violated values is something that is achieved with others and not by oneself (Wenzel et al. 2021). Offenders who co-reflect with victims may therefore be more likely to meta-perceive value consensus than instances where victims only engage in individual reflection. Put simply, offenders may be more likely to believe that the victim’s forgiveness is genuine after co-reflection, and that it is not pseudo-forgiveness, because the forgiveness is seen to be a result of their joint working through of the issue.

## The Present Studies

We report two studies that investigated offenders’ appraisals of victims’ forgiveness as a function of engagement. In Study 1, we presented participants with vignettes that contained instructions to imagine committing a wrongdoing. We then manipulated the level of engagement that followed the transgression and preceded the expression of forgiveness. In Study 2, we investigated real experiences of interpersonal offences. We recruited participants on the basis that they had recently wronged somebody and asked them for their assessment of the level of engagement that had occurred between them and the victim, and how they perceived the victim’s forgiveness.

We report all manipulations, and exclusions from both studies. Both studies were preregistered including study design, hypotheses, measures, power analyses, inclusion/exclusion criteria, and pre-planned primary analyses. The Human Research Ethics Committee at Flinders University approved this research (Approval Code 7991). All participants provided informed consent. These pre-registrations along with the data and materials for both studies can be accessed via the Open Science Framework link: https://osf.io/vbw7a/?view_only=7864f1f11003478eaecc3be02a0db554 (Study 1); https://osf.io/rxfcp/?view_only=7a725b7024db4883a4e70ffa41fbc36f (Study 2).

### Study 1

Study 1 was an experiment in which we asked participants to imagine committing a wrongdoing. We constructed five scenarios that each included a different relationship (e.g., friend, partner), and type of wrongdoing (e.g., betrayal of trust, destruction of property) to reinforce the generalisability of findings.^1^ Following the scenario, participants were randomly allocated to one of three levels of engagement with the victim (i.e., co-reflection, individual reflection, and no reflection).

We predicted:

**(1a)** Co-reflection (vs. no reflection) would lead to greater attributions of processing; (1b) Co-reflection (vs. no reflection) would be negatively related to perceived avoidance via processing (indirect effect); (1c) Co-reflection (vs. no reflection) would be negatively related to perceived minimisation via processing (indirect effect).
**(2a)** Individual reflection (vs. no reflection) would lead to greater attributions of processing; (2b) Individual reflection (vs. no reflection) would be negatively related to perceived avoidance via processing (indirect effect); (2c) Individual reflection (vs. no reflection) would be negatively related to perceived minimisation via processing (indirect effect).
**(3a)** Co-reflection (vs. no reflection) would lead to greater meta-perceived value consensus; (3b) Co-reflection (vs. no reflection) would be positively related to perceived genuine forgiveness via meta-perceived value consensus (indirect effect); (3c) Co-reflection (vs. no reflection) would be negatively related to perceived unforgiveness via meta-perceived value consensus (indirect effect).
**(4a)** Co-reflection (vs. individual reflection) would lead to greater meta-perceived value consensus; (4b) Co-reflection (vs. individual reflection) would be positively related to perceived genuine forgiveness via meta-perceived value consensus (indirect effect); (4c) Co-reflection (vs. individual reflection) would be negatively related to perceived unforgiveness via meta-perceived value consensus (indirect effect).

In Study 1, we also had a number of exploratory interests. One of these related to whether the victim’s co-reflection with the offender might lead offenders to perceive the victim as having considered the *whole story*; that is, as having comprehensively assessed the incident by considering different views of the incident and the offender’s feelings. We report here the results for whole story because it showed promising relationships to our outcome variables that we then subsequently decided to follow up in Study 2. However, we report the exploratory analyses for other variables in the Online Material.

#### Method

##### Participants

We conducted a Monte Carlo power analysis for indirect effects with parameters set following guidelines from Schoemann et al. (2017). Our population parameters for the model were determined with the correlation matrix and standard deviations option utilising *r* = .20 as the expected correlation coefficient between predictor, mediator, and outcome given the median effect size is *r* = .24 in typical social psychological studies (Lovakov & Agadullina 2021). The power analysis revealed that approximately 309 participants were required to achieve 80% statistical power for detecting the hypothesised indirect effect. Data from one participant were removed due to not completing all the measures. Participants were recruited via the research platform Prolific and were residents of the United Kingdom (225 female, 82 male, 1 non-binary; *M*_age_ = 42.2); 92.9% were White/White British, 3.6% Asian/Asian British, 2.3% multiracial, 1% Black/African/Caribbean British, and 0.3% Middle Eastern.

##### Design and Procedure

We randomly allocated participants to 1 of 5 possible wrongdoing scenarios. These included taking a trip and missing a meeting to work on a shared assignment, sleeping in and missing breakfast with an old friend, getting drunk and kissing someone who was not your partner, knocking over a vase belonging to your extended family, and disclosing a friend’s secret to a group. Following the wrongdoing scenario, participants were then randomly allocated to an engagement condition: no reflection, individual reflection, or co-reflection. In all conditions the participants were asked to imagine that they had reached out to the victim, and the victim replied either that they did not wish to think about it (no reflection), they wished to think about it alone (individual reflection) or wished to talk with the participant (co-reflection). At the end of the scenario, in all conditions the participants read that the victim had subsequently forgiven them. The complete instructions for these conditions are available via this OSF link (https://osf.io/vbw7a/?view_only=7864f1f11003478eaecc3be02a0db554). Following the manipulation, participants responded to the measures and were debriefed.

##### Measures

All items were measured on a 7-point Likert scale (1 = strongly disagree, 2 = disagree, 3 = somewhat disagree, 4 = neither agree nor disagree, 5 = somewhat agree, 6 = agree, 7= strongly agree), unless otherwise indicated. Items were averaged on all measures to create a single score. All measures and the manipulation were constructed for the present research. The complete item list for each scale is viewable via the OSF link provided.

***Avoidance.*** A five-item scale measured the extent to which participants perceived the victim to be avoiding the incident (e.g., ‘I believe this person just wants to forget what happened’; α = 0.88).

***Minimisation.*** A five-item scale measured the extent to which participants perceived the victim to be trying to persuade themselves that they were unaffected by the incident (e.g., ‘I believe this person is trying to convince themself that it did not bother them’; α = 0.89).

***Genuine Forgiveness.*** A five-item scale measured the extent to which participants perceived the victim to hold true forgiving sentiment (e.g., ‘I believe this person genuinely forgives me for what I did’; α = 0.88).

***Unforgiveness***. A five-item scale measured the extent to which participants perceived the victim to hold an unforgiving sentiment (e.g., ‘I believe this person still harbors a grudge against me’; α = 0.83).

***Processing.*** A three-item scale measured the extent to which participants perceived the victim had engaged in effortful processing of the incident (e.g., ‘I believe this person has spent time working through what happened’; α = 0.91).

***Meta-Perceived Value Consensus.*** A five-item scale measured the extent to which participants perceived that the victim shared a value consensus with them (e.g., ‘This person believes that I am still committed to the important values in our relationship’; α = 0.93).

***Whole Story.*** A three-item scale measured the extent to which participants perceived the victim had taken a comprehensive view of the incident (e.g., ‘I believe this person has taken into account all sides of the story’; α = 0.93).

#### Results

series of One-Way ANOVAs were used to analyse the effect of reflection condition on all dependent variables.^2^ Table 1 presents the descriptive statistics, *F*-tests, and effect sizes. All omnibus tests were significant. Therefore, we conducted a Tukey post-hoc test for minimisation, but Games-Howell post-hoc tests were conducted for all other dependent variables due to violations of the homogeneity of variance assumption.

**Table 1:** Descriptive and Inferential Statistics as a Function of Reflection Condition with Omnibus and Post-Hoc Tests (Study 1).


	REFLECTION CONDITION					CONTRAST 1 CR vs IR		CONTRAST 2 CR vs NR		CONTRAST 3 IR vs NR	

VARIABLE	CO-REFLECTION (*N* = 102) *M* (*SD*)	INDIVIDUAL REFLECTION (*N* = 103) *M* (*SD*)	NO REFLECTION (*N* = 103) *M* (*SD*)	*F*(2, 305)	η^2^	*T*	*D*	*T*	*D*	*T*	*D*

1. Avoidance	3.18 (1.15)	3.38 (1.13)	4.70 (1.07)	56.0^***^	0.27	–1.22	0.17	–9.76^***^	1.36	–8.61^***^	1.18

2. Minimisation	3.18 (1.17)	3.37 (1.13)	4.20 (1.15)	22.6^***^	0.13	–1.17	0.16	–6.32^***^	0.88	–5.16^***^	0.72

3. Genuine Forgiveness	5.59 (0.75)	5.38 (0.75)	5.00 (1.06)	12.3^***^	0.08	2.02	0.24	4.62^***^	0.68	2.97^**^	0.44

4. Unforgiveness	2.57 (0.86)	3.11 (0.95)	3.25 (1.10)	13.7^***^	0.08	–4.25^***^	0.55	–4.90^***^	0.69	–0.98	0.14

5. Processing	5.85 (0.73)	5.93 (0.68)	5.13 (1.27)	22.9^***^	0.13	–0.87	0.09	4.95^***^	0.77	5.65^***^	0.86

6. Meta-Perceived VC	5.38 (0.93)	5.15 (0.97)	4.80 (1.13)	8.45^***^	0.05	1.73	0.23	4.00^***^	0.57	2.37^*^	0.34

7. Whole Story	5.61 (0.86)	5.22 (1.10)	4.63 (1.33)	20.3^***^	0.12	2.87^*^	0.35	6.29^***^	0.88	3.46^**^	0.53


*Note*. *p < .05, **p < .01, ***p < .001. CR = Co-Reflection, IR = Individual Reflection, NR = No Reflection, VC = Value Consensus.

As predicted, co-reflection (H1a), and individual reflection (H2a), led to greater perceived processing, than no reflection. Also, in line with predictions, co-reflection led to greater meta-perceived value consensus than no reflection (H3a). However, contrary to predictions, co-reflection did not lead to greater meta-perceived value consensus than individual reflection (H4a). Hence, the subsequent indirect effects hypotheses were not supported (H4b/H4c).

##### Mediation Analyses

**Figure 1:**
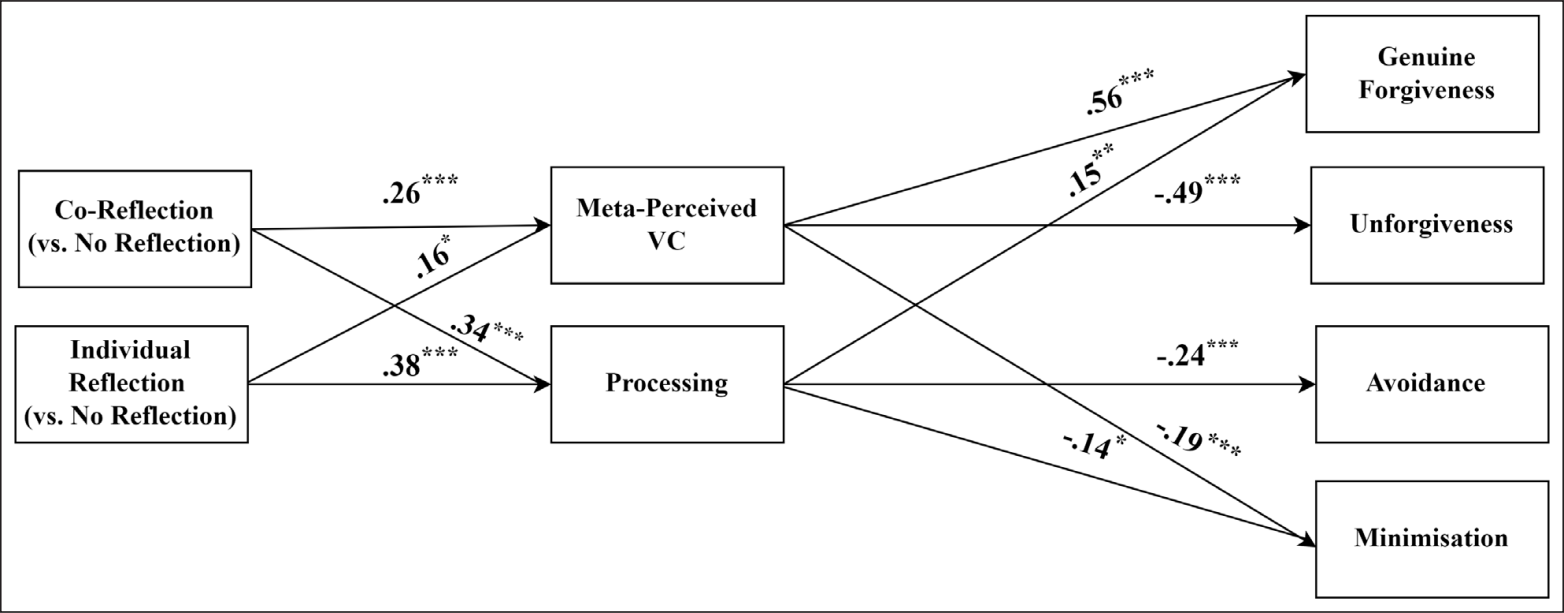
Standardised coefficients for the Mediated Regression Analyses of the Effect of Co-Reflection (vs. no reflection) and Individual Reflection (vs. no reflection) on the Meanings of Forgiveness via the Key Mediators (Study 1). *Note*. *p < .05, **p < .01, ***p < .001. These results are summarised from separate analyses.

Table 2 contains the bivariate correlations between all outcome variables. We conducted a parallel mediation regression analysis for each outcome variable using Process Model 4 (95% percentile bootstraps set at 5,000; Hayes, 2017) to test our indirect effect hypotheses. We specified a multicategorical predictor variable; the co-reflection and individual reflection conditions were each represented by a dummy variable, with the no-reflection control group serving as shared reference condition. We included both processing and meta-perceived value consensus as mediators in each model. The top panel in Table 3 presents the unstandardised effects of the predictor variables onto the two mediators (being the same for all four mediation models); the bottom panel shows the effects of predictors and mediators onto the four outcome variables. Figure 1 presents the standardised coefficients for these pathways. For economy, we present the results for the four outcome variables together despite these coming from separate analyses.

**Table 2:** Bivariate Correlations for Main Variables (Study 1).


VARIABLE	1	2	3	4	5	6	7

1. Avoidance	–	.64^***^	–.20^***^	.29^***^	–.38^***^	–.17^**^	–.37^***^

2. Minimisation		–	–.33^***^	.49^***^	–.32^***^	–.32^***^	–.40^***^

3. Genuine Forgiveness			–	–67^***^	.42^***^	.65^***^	.51^***^

4. Unforgiveness				–	–.31^***^	–.56^***^	–.50^***^

5. Processing					–	.43^***^	.57^***^

6. Meta-Perceived VC						–	.59^***^

7. Whole Story							–


*Note*. *p < .05, **p < .01, ***p < .001.

**Table 3:** Unstandardised Coefficients for Predictors on Mediators, and Coefficients for the Predictors and Mediators on Outcome Variable.


*DEPENDENT VARIABLE*	*B*	*SE*	*T*	CI_95%_	*DEPENDENT VARIABLE*	*B*	*SE*	*T*	CI_95%_

*Processing*	*R^2^* = .13, *F*(2,305) = 22.9, *p* < .001				*Meta-Perceived VC*	*R^2^* = .05, *F*(2,305) = 8.45, *p* < .001			

Co-Reflection	.72	.13	5.50	[0.46, 0.97]	Co-Reflection	.58	.14	4.08	[0.30, 0.85]

Individual Reflection	.80	.13	6.17	[0.55, 1.06]	Individual Reflection	.35	.14	2.47	[0.07, 0.62]

*Model 1: Avoidance*	*R^2^* = .31, *F*(4,303) = 34.5, *p* < .001				*Model 2: Minimisation*	*R^2^* = .20, *F*(4,303) = 19.2, *p* < .001			

Co-Reflection	–1.32	.16	–8.21	[–1.63, –1.00]	Co-Reflection	–.76	.16	–4.65	[–1.08, –0.44]

Individual Reflection	–1.09	.16	–6.77	[–1.40, –0.77]	Individual Reflection	–.61	.16	–3.71	[–0.93, –0.29]

Processing	–.31	.07	–4.23	[–0.45, –0.16]	Processing	–.18	.07	–2.38	[–0.32, –0.03]

Meta-Perceived VC	.03	.07	0.50	[–0.10, 0.17]	Meta-Perceived VC	–.23	.07	–3.36	[–0.36, –0.10]

*Model 3: Genuine Forgiveness*	*R^2^* = .45, *F*(4,303) = 63.2, *p* < .001				*Model 4: Unforgiveness*	*R^2^* = .35, *F*(4,303) = 40.2, *p* < .001			

Co-Reflection	.22	.09	2.18	[0.02, 0.41]	Co-Reflection	–.34	.12	–2.77	[–0.58, –0.10]

Individual Reflection	.10	.10	1.06	[–0.09, 0.30]	Individual Reflection	.10	.12	0.79	[–0.14, 0.34]

Processing	.13	.05	2.96	[0.04, 0.22]	Processing	–.09	.06	–1.58	[–0.20, 0.02]

Meta-Perceived VC	.49	.04	11.9	[0.41, 0.57]	Meta-Perceived VC	–.48	.05	–9.44	[–0.58, –0.38]


*Note*. No-reflection is the reference condition. The bottom panel contains four models (separate analyses).

Table 4 displays all the pre-registered indirect effects that contrasted co-reflection to no reflection and individual reflection to no reflection. The residual direct effects were significant indicating partial mediation. For the contrasts between co-reflection and no reflection, as predicted (H1b), co-reflection (vs. no reflection) had a significant negative indirect effect on avoidance, via processing. Also as predicted (H1c), co-reflection (vs. no reflection) had a significant negative indirect effect on minimisation, via processing. As predicted (H3b), co-reflection (vs. no reflection) had a significant positive indirect effect on genuine forgiveness, via meta-perceived value consensus. Further, as predicted (H3c), co-reflection (vs. no reflection) had a significant negative indirect effect on unforgiveness, via meta-perceived value consensus.

**Table 4:** Bias-Corrected Bootstrap Estimates for Indirect Effects (Study 1).


*DEPENDENT VARIABLE*	*IE*	*SE*	CI_95%_	*DEPENDENT VARIABLE*	*IE*	*SE*	CI_95%_

*Avoidance*				*Minimisation*			

CR → Processing	–.22	.07	[–0.37, –0.10]	CR → Processing	–.13	.07	[–0.27, –0.01]

IR → Processing	–.25	.08	[–0.41, –0.11]	IR → Processing	–.14	.08	[–0.30, –0.01]

*Genuine Forgiveness*				*Unforgiveness*			

CR → Meta-Perceived VC	.28	.08	[0.14, 0.45]	CR → Meta-Perceived VC	–.28	.08	[–0.43, –0.14]


*Note*. CR = co-reflection, IR = individual reflection.

The contrasts between individual reflection and no reflection were also as predicted. Individual reflection (vs. no reflection) had a significant negative indirect effect on avoidance, via processing (H2b). Finally, individual reflection (vs. no reflection) had a significant negative indirect effect on minimisation, via processing (H2c).

##### Exploratory analyses

Our exploratory analyses^3^ also indicated that co-reflection led to a greater perception that the victim had considered the whole story than both individual reflection and no reflection conditions, but individual reflection also led to greater whole story perceptions than no reflection. We thus conducted exploratory indirect effect analyses with whole story as the mediator. In these analyses, we entered whole story as a single mediator. We present the full results in the Online Material, but co-reflection (vs. no reflection) and individual reflection (vs. no reflection) had significant negative indirect effects on avoidance, minimisation, and unforgiveness, via whole story. Finally, co-reflection (vs. no reflection) and individual reflection (vs. no reflection) had significant positive indirect effects on genuine forgiveness, via whole story.

#### Discussion

Study 1 provided evidence that both co-reflection and individual reflection have direct causal effects, and indirect effects, on offenders’ appraisals of victims’ expressed forgiveness. In line with predictions, co-reflection (vs. no reflection) and individual reflection (vs. no reflection) led to greater perceptions of processing. Directly, and through processing, co-reflection (vs. no reflection) and individual reflection (vs. no reflection) reduced interpretations of avoidance and minimisation. Moreover, co-reflection (vs. no reflection) caused offenders to perceive that the victim shared a value consensus with them (i.e., meta-perceived value consensus). Directly, and via meta-perceived value consensus, co-reflection (vs. no reflection) reduced appraisals of unforgiveness and increased perceptions of genuine forgiveness. However, contrary to predictions, co-reflection did not lead to greater meta-perceived value consensus than individual reflection.

Our exploratory analyses suggested that whole story may be a key consideration for how offenders appraise victims’ forgiveness. Co-reflection caused offenders to perceive that the victim had considered the whole story over and above individual reflection and no reflection. Moreover, co-reflection (vs. no reflection) and individual reflection (vs. no reflection) were positively related to genuine forgiveness, and negatively related to avoidance, minimisation, and unforgiveness, via whole story. These findings are consistent with previous research that during interpersonal conflict if one believes that the other person takes their point of view and understands their thoughts and feelings, then they believe that the relationship is in a more positive state (Gordon & Chen 2016). An offender believing the victim has considered the whole story leads them to appraise the victim as having arrived at their response in a more considered way, indicating genuineness in their forgiveness and lack of minimisation or avoidance.

A limitation of this study was the use of vignettes which presents a limitation to the ecological validity of the findings despite the various wrongdoings and relationships with the victim we provided participants to enhance generalisability. Accordingly, we conducted Study 2 to examine real experiences of committing wrongdoing, and how offenders appraise forgiveness based on their assessment of the level of co-reflection with the victim, and individual reflection by the victim.

### Study 2

We used a recall design for Study 2 to capture real experiences of committing wrongdoing and post-transgression engagement. Thus, co-reflection, and individual reflection were measured variables in this study, rather than manipulated variables. We asked participants to recall a recent wrongdoing they had committed against somebody they shared a relationship with and then rate the level of co-reflection they had with the victim about the issue, and how much individual reflection about the issue they perceived the victim to have engaged in.

Our first three sets of predictions remained unchanged from Study 1 except that co-reflection and individual reflection were now continuous variables rather than two experimental conditions compared against a control group.^4^ The original fourth set of predictions, which entailed a comparison between experimental conditions, likewise did not apply and was therefore dropped. However, we included a new set of pre-registered predictions for whole story, attempting to replicate the exploratory findings from Study 1. Specifically, (5a) Co-reflection would be positively related to whole story; (5b) Co-reflection would be positively related to perceived genuine forgiveness via whole story (indirect effect).

We also pre-registered exploratory moderation predictions. Specifically, we anticipated that whether the victim had indicated forgiveness (or not) would moderate the second leg of the predicted mediations. In particular, if the victim had indicated forgiveness, then processing would be negatively related to minimisation, and avoidance. Also, if the victim had indicated forgiveness, then whole story, and meta-perceived value consensus, would be positively related to genuine forgiveness.

#### Method

##### Participants

We conducted a Monte Carlo power analysis for indirect effects (Schoemann et al. 2017). Our population parameters for the model were the correlations from Study 1 for the co-reflection to genuine forgiveness via meta-perceived value consensus model. This revealed that approximately 186 participants were required to achieve 80% statistical power for detecting the hypothesised indirect effect, but we requested data from 200 participants to account for any data exclusions. Indeed, we retained 179 participants after excluding data from 4 participants who failed more than one attention check, and data from 17 participants who failed to describe an instance of committing a wrongdoing when prompted to in an open response question. Participants were recruited from Amazon Mechanical Turk via the platform CloudResearch and were residents of the United States (91 male, 87 female, 1 non-binary; *M*_age_ = 39.2); 73.7% were White, 11.2% Black/African American, 7.3% Asian, 4.5% Hispanic or Latino, and 3.4% multiracial.

##### Design and Procedure

We pre-registered our inclusion criteria and screened accordingly. First, our participants must have seriously wronged somebody they share a relationship with, as trivial wrongdoings may not require any form of redress. We screened for this requirement with a single item: ‘How serious do you believe your wrongdoing was?’ (1 = not at all; 4 = moderately; 7 = extremely). The survey directed sign-ups to the end of survey if they indicated the severity was below the mid-point of the scale (i.e., <moderately severe). Second, the victimised person must have been aware of the wrongdoing as a lack of awareness precludes any engagement. Third, the wrongdoing must have occurred at least a week ago, to allow some time for engagement, but no longer than a month ago, to retain some recency of the incident and participants’ recall of it.

Following screening, we asked eligible sign-ups to describe their wrongdoing, rate the intentionality of their wrongdoing (*M* = 4.77, *SD* = 2.00), the perceived forgivability of their wrongdoing (*M* = 4.52, *SD* = 1.55), categorise the type of wrongdoing (49.2% betrayal of trust, 18.4% other, 16.8% insult, 7.8% infidelity, 6.1% betrayal of confidence, and 1.7% physical abuse/intentional harm), and indicate what their relationship was with the victim (36.3% significant other, 26.8% family member, 20.7% close friend, 10.1% work colleague, 3.4% other, and 2.8% acquaintance). Participants then responded to a single item that asked whether they had received some indication of forgiveness from the victim (i.e., ‘Has the other person claimed or indicated in any way that they forgive you?’; 51.4% indicated no forgiveness, 48.6% indicated forgiveness).

##### Measures

All items were measured on a 7-point Likert scale (1 = strongly disagree, 2 = disagree, 3 = somewhat disagree, 4 = neither agree nor disagree, 5 = somewhat agree, 6 = agree, 7= strongly agree), unless otherwise indicated. Items were averaged on all measures to create a single score. We included all measures from Study 1: Avoidance (α = 0.91); Minimisation (α = 0. 94); Genuine Forgiveness (α = 0.96); Unforgiveness (α = 0.89); Processing (α = 0.92); Meta-Perceived Value Consensus (α = 0.96); Whole Story (α = 0.94). We included two additional measures to capture co-reflection and individual reflection.

***Co-Reflection.*** We used the five-item co-reflection scale from the Transgression-Related Co-Rumination Scale (Thai et al., 2023) to measure co-reflection (e.g., ‘This person and I seemed to make good progress in talking about the incident’; α = 0.96).

***Individual Reflection.*** A five-item scale measured participants’ perceived level of individual reflection that the victim had engaged in (e.g., ‘I believe this person spent time thinking privately about the incident’; α = 0.89).

#### Results

Table 5 displays the means, standard deviations, and bivariate zero-order correlations. We used a series of separate parallel mediation analyses using Process Model 4 and Process Model 14 to test both our indirect effect predictions and to explore possible moderated mediation by indicated forgiveness. We used bootstraps set at 5,000 replications to estimate 95% percentile confidence intervals. To test our exploratory moderation hypotheses, we calculated conditional effects at the –1SD and +1SD to represent low and high levels of the moderator. Both predictors, and all pre-registered mediators (i.e., processing, meta-perceived value consensus, and whole story) were included in each analysis.

**Table 5:** Means, Standard Deviations, and Bivariate Correlations for Main Variables (Study 2).


VARIABLE	*M* (*SD*)	1	2	3	4	5	6	7	8	9

1. Co-Reflection	4.64 (1.72)	–	.14	.25^***^	.20^**^	.78^***^	–.54^***^	.41^***^	.77^***^	.70^***^

2. Individual Reflection	5.86 (0.96)		–	–.10	–.10	.14	–.06	.58^***^	.11	.31^***^

3. Avoidance	3.58 (1.51)			–	.75^***^	.31^***^	.08	–.01	.23^**^	.09

4. Minimisation	3.15 (1.54)				–	.17^*^	.14	–.07	.11	.07

5. Genuine Forgiveness	4.75 (1.70)					–	–.63^***^	.34^***^	.80^***^	.66^***^

6. Unforgiveness	3.51 (1.58)						–	–.08	–.53^***^	–.45^***^

7. Processing	5.28 (1.33)							–	.46^***^	.47^***^

8. Meta-Perceived VC	4.89 (1.65)								–	.64^***^

9. Whole Story	4.83 (1.55)									–


*Note*. *p < .05, **p < .01, ***p < .001. VC = Value Consensus.

**Figure 2:**
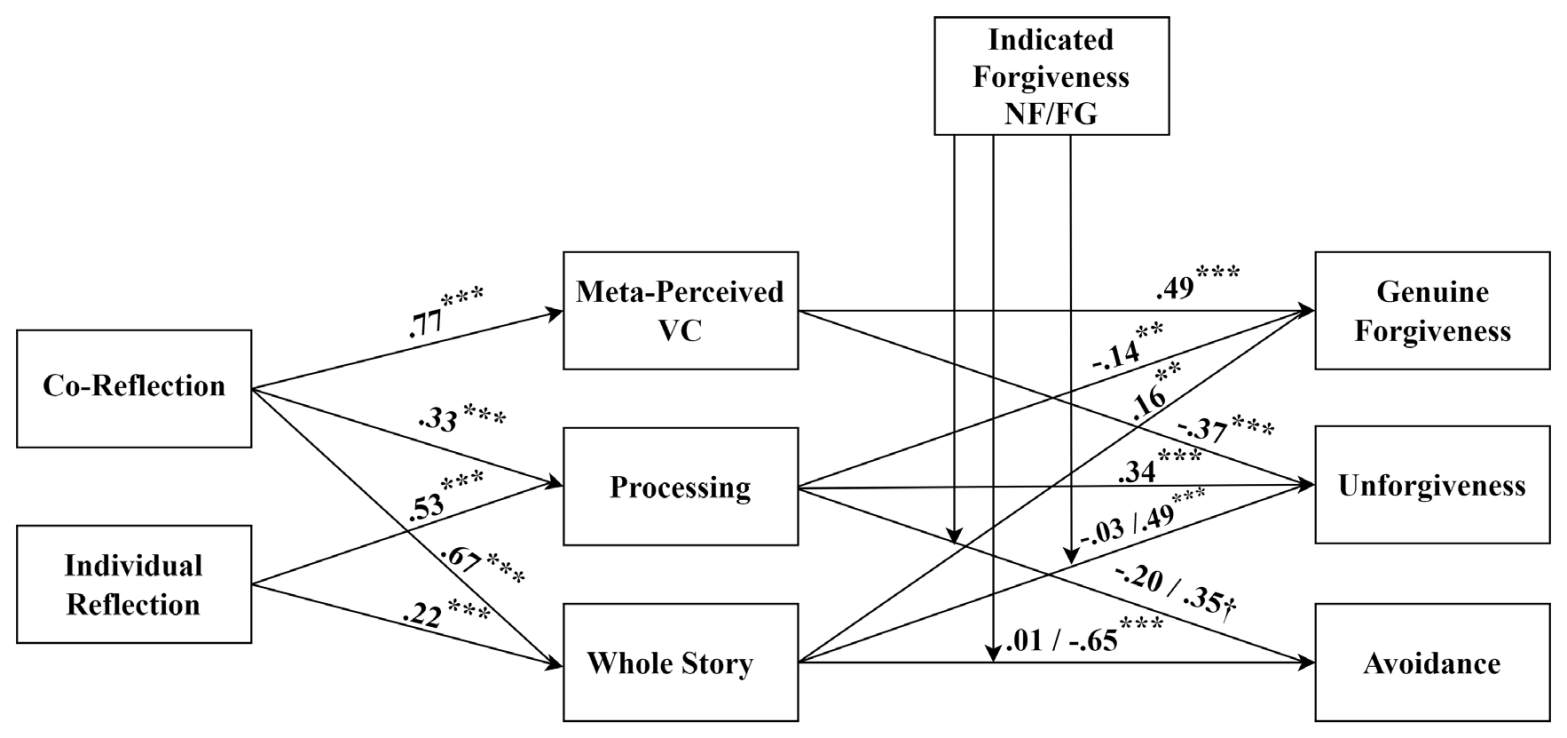
Standardised coefficients for the Mediated Regression Analyses of the Effect of Co-Reflection and Individual Reflection on the Meanings of Forgiveness via the Key Mediators with Moderation by Indicated Forgiveness (Study 2). *Note*. ^†^*p* = .05, *p < .05, **p < .01, ***p < .001. NF = no indicated forgiveness, FG = indicated forgiveness. These results are summarised from separate analyses.

We report the unstandardised coefficients for the predictor-mediator pathways in Table 6. Figure 2 displays the standardised coefficients for all pathways, but the results for the mediator-outcome variables are from separate analyses. For conciseness, our reporting of the findings focuses on the pre-registered predictions. As predicted, co-reflection (H1a) and individual processing (H2a) were positively related to processing. Co-reflection was also positively related to meta-perceived value consensus (H3a) and whole story (H5a), as predicted.

**Table 6:** Unstandardised Coefficients for Predictors on Mediators (Study 2).


*DEPENDENT VARIABLE*	*B*	*SE*	*T*	CI_95%_

*Processing*	*R^2^* = .44, *F*(2,176) = 69.3, *p* < .001			

Co-Reflection	.26	.04	5.84	[0.17, 0.34]

Individual Reflection	.73	.08	9.29	[0.58, 0.89]

*Meta-Perceived Value-Consensus*	*R^2^* = .59, *F*(2,176) = 126.5, *p* < .001			

Co-Reflection	.74	.05	15.7	[0.65, 0.83]

Individual Reflection	.01	.08	0.11	[–0.16, 0.17]

*Whole Story*	*R^2^* = .54, *F*(2,305) = 102.8, *p* < .001			

Co-Reflection	.61	.05	13.0	[0.51, 0.70]

Individual Reflection	.35	.08	4.23	[0.19, 0.52]


Table 7 summarises the unstandardised coefficients for the mediator-outcome pathways from separate analyses. Contrary to our indirect effect predictions (H1b/H2b), processing was not a significant negative predictor of avoidance. Also contrary to our predictions, processing was not a significant negative predictor of minimisation (H1c/H2c). However, meta-perceived value consensus, and whole story, were positively related to genuine forgiveness, and negatively related to unforgiveness.

**Table 7:** Unstandardised Coefficients of the Mediators and Moderator (Indicated Forgiveness) on Dependent Variables (Study 2).


*DEPENDENT VARIABLE*	*B*	*SE*	*T*	CI_95%_	*DEPENDENT VARIABLE*	*B*	*SE*	*T*	CI_95%_

*Model 1: Avoidance*	*R^2^* = .16, *F*(9, 169) = 3.61, *p* < .001				*Model 2: Minimisation*	*R^2^* = .10, *F*(9, 169) = 2.20, *p* < .001			

Co-Reflection	.26	.13	2.03	[0.01, 0.52]	Co-Reflection	.29	.14	2.14	[0.02, 0.56]

Individual Reflection	–.22	.15	–1.46	[–0.51, 0.08]	Individual Reflection	–.10	.16	–0.65	[–0.41, 0.21]

Processing	.09	.13	0.69	[–0.17, 0.35]	Processing	–.07	.14	–0.48	[–0.34, 0.21]

Meta-Perceived VC	.15	.12	1.29	[–0.08, 0.38]	Meta-Perceived VC	–.06	.12	–0.52	[–0.31, 0.18]

Whole Story	–.32	.12	–2.65	[–0.56, –0.08]	Whole Story	–.17	.13	–1.34	[–0.42, 0.08]

Indicated Forgiveness	–.07	.33	–0.21	[–0.73, 0.58]	Indicated Forgiveness	.03	.35	0.09	[–0.66, 0.72]

*Model 3: Genuine Forgiveness*	*R^2^* = .73, *F*(9,169) = 51.0, *p* < .001				*Model 4: Unforgiveness*	*R^2^* = .47, *F*(9,169) = 16.8, *p* < .001			

Co-Reflection	.28	.08	3.45	[0.12, 0.44]	Co-Reflection	–.09	.11	–0.83	[–0.30, 0.12]

Individual Reflection	.12	.09	1.21	[–0.07, 0.30]	Individual Reflection	–.23	.12	–1.90	[–0.48, 0.01]

Processing	–.20	.08	–2.35	[–0.36, –0.03]	Processing	.46	.11	4.24	[0.25, 0.67]

Meta-Perceived VC	.51	.07	6.81	[0.36, 0.66]	Meta-Perceived VC	–.34	.10	–3.55	[–0.53, –0.15]

Whole Story	.23	.08	2.97	[0.08, 0.38]	Whole Story	–.27	.10	–2.67	[–0.46, –0.07]

Indicated Forgiveness	.23	.21	1.10	[–0.18, 0.65]	Indicated Forgiveness	–.81	.27	–2.97	[–1.36, –0.27]


*Note*. VC = value consensus, interaction terms are omitted, indicated forgiveness coded: 1 = no indication, 2 = forgiveness indication.

##### Exploratory Moderation by Indicated Forgiveness

**Figure 3:**
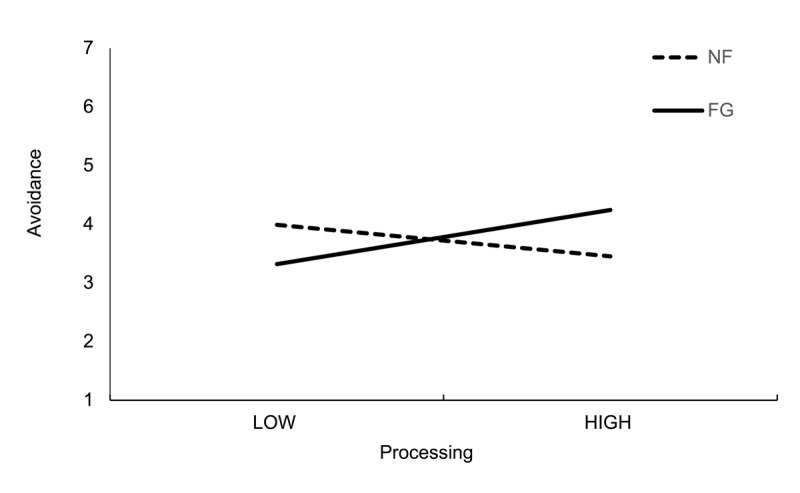
The relationship between processing and avoidance as a function of indicated forgiveness (Study 2). NF = no indicated forgiveness, FG = victim indicated forgiveness.

Indicated forgiveness moderated the effects of some mediator-outcome pathways. For example, indicated forgiveness significantly moderated the relationship between processing and avoidance. Table 8 contains the indices of moderated mediation. Figure 2 displays the standardised coefficients for the moderating effects. Different from our pre-registered speculation, when the victim had *not* indicated forgiveness, there was a negative relationship (but non-significant simple effect) between processing and avoidance, whereas there was a *positive* relationship (with a marginally significant simple effect) between processing and avoidance when the victim had indicated forgiveness (see Figure 3).

**Table 8:** Bias-Corrected Bootstrap Estimates for Simple Mediation, Moderated Mediation, and Conditional IE by Indicated Forgiveness.


*DEPENDENT VARIABLE*	SIMPLE MEDIATION			MODERATED MEDIATION			CONDITIONAL INDIRECT EFFECTS					

	IE	SE	CI_95%_	Index	SE	CI_95%_	NF	SE	CI_95_%	FG	SE	CI_95_%

*Avoidance*												

CR → Processing	–.02	.04	[–0.11, 0.06]	.16	.07	[0.04, 0.30]	–.06	.04	[–0.15, 0.02]	.10	.06	[–0.01, 0.22]

IR → Processing	–.07	.11	[–0.29, 0.16]	.46	.17	[0.12, 0.80]	–.17	.13	[–0.43, 0.06]	.29	.15	[–0.01, 0.58]



*Minimisation*												

CR → Processing	–.04	.03	[–0.09, 0.03]	.02	.06	[–0.11, 0.14]	–.03	.03	[–0.09, 0.04]	–.01	.06	[–0.13, 0.10]

IR → Processing	–.10	.09	[–0.29, 0.07]	.06	.17	[–0.28, 0.40]	–.08	.10	[–0.31, 0.09]	–.02	.16	[–0.35, 0.29]



*Genuine Forgiveness*												

CR → Meta-Perceived VC	.37	.07	[0.23, 0.50]	.03	.13	[–0.22, 0.31]	.36	.09	[0.16, 0.51]	.39	.11	[0.18, 0.59]

CR → Whole Story	.11	.05	[–0.01, 0.21]	.13	.11	[–0.10, 0.33]	.07	.06	[–0.05, 0.20]	.20	.10	[0.01, 0.36]



*Unforgiveness*												

CR → Meta-Perceived VC	–.26	.08	[–0.40, –0.10]	–.04	.15	[–0.38, 0.20]	–.24	.08	[–0.39, –0.06]	–.27	.13	[–0.58, –0.07]

CR → Whole Story	–.08	.06	[–0.19, 0.03]	–.28	.12	[–0.49, –0.02]	–.02	.06	[–0.14, 0.10]	–.30	.10	[–0.48, –0.06]


*Note*. IE = indirect effect, CR = co-reflection, IR = individual reflection, VC = value consensus, NF = no indicated forgiveness, FG = indicated forgiveness.

##### Indirect Effects

Table 8 displays the indirect effects, and conditional indirect effects by indicated forgiveness. The conditional indirect effects of co-reflection and individual reflection on avoidance via processing reflected the interactions detailed above. That is, the conditional indirect effect was negative when the victim had not indicated forgiveness, but *positive* when the victim had indicated forgiveness.

The indirect effects of co-reflection on genuine forgiveness were not conditional on indicated forgiveness. As predicted (H3b), co-reflection had a significant positive indirect effect on genuine forgiveness via meta-perceived value consensus (partial mediation). However, support for H5b was only marginal, as the indirect effect of co-reflection on genuine forgiveness via whole story was only marginally significant.

For unforgiveness, there was a significant negative indirect effect of co-reflection via meta-perceived value consensus (partial mediation), and no moderating effect of indicated forgiveness. Finally, the indirect effect of co-reflection on unforgiveness via whole story was moderated by indicated forgiveness: there was no conditional indirect effect when the victim had not indicated forgiveness, but significant negative conditional indirect effect when the victim had indicated forgiveness.

#### Discussion

Study 2 provided further evidence that engagement following wrongdoing affects how offenders appraise victims’ forgiveness. Yet, in this study, only co-reflection was positively related to meta-perceived value consensus. Importantly, co-reflection facilitated perceptions of genuine forgiveness, via meta-perceived value consensus, and, marginally, via whole story. Additionally, co-reflection led offenders to perceive lower unforgiveness via meta-perceived value consensus.

Like Study 1, both co-reflection and individual reflection were positively related to processing. However, there was an unexpected lack of indirect effects of co-reflection and individual reflection on avoidance and minimisation, via processing. Yet, processing was associated with greater perceptions of avoidance, but only when the victim had indicated forgiveness. It is possible that offenders might see victims high in processing but ostensibly forgiving as being more avoidant because these victims are trying to cut short their own thinking about the issue (e.g., attempting thought suppression or cognitive avoidance).

There were also some inconsistencies between the two studies. In contrast to Study 1, there were no significant indirect effects of co-reflection or individual reflection on minimisation via processing. Also, both avoidance and minimisation were positively associated with co-reflection, and (at the level of zero-order correlations) with genuine forgiveness. This may reflect the ambiguity of avoidance and minimisation; it could be that offenders believe the victim is trying to move past the incident or downplay its significance because they are forgiving (Waldron & Kelley 2005). Other research suggests that characteristics of the relationship and the transgression may shape why victims avoid or minimise transgressions (Guerrero & Bachman 2010). Future research could further explore how these contextual variables also shape how offenders make sense of or give meaning to victims’ avoidance and/or minimisation.

## General Discussion

Forgiveness is important in the moral repair process, but forgiveness can mean different things. A victim could be sincere by offering genuine forgiveness to the offender, or they may be less sincere – it could be pseudo-forgiveness. Offenders who receive forgiveness do not have direct knowledge of what victims mean when they forgive; they do not have insight into victims’ true sentiments. Instead, offenders must appraise the meaning of the forgiveness offered to them (Gollwitzer & Okimoto 2021). In these studies, we investigated how offenders appraise victims’ forgiveness based on the level of engagement that occurs in the aftermath of the wrongdoing and precedes the expression of forgiveness. We found that appraisals of forgiveness are shaped by dyadic dynamics unfolding after a transgression.

The present studies provide evidence that co-reflection is a more consistent driver than individual reflection for offenders to perceive genuine forgiveness and to perceive lower unforgiveness. Study 1 provided causal evidence that co-reflection (vs. no reflection) and individual reflection (vs. no reflection) led to greater perceived genuine forgiveness, but co-reflection led to lower perceived unforgiveness than both individual reflection and no reflection. Further, only co-reflection was a consistent significant predictor of meta-perceived value consensus in both studies; and only co-reflection had significant indirect effects on genuine forgiveness and reduced unforgiveness via meta-perceived value consensus in both studies. In Study 1, both individual reflection and co-reflection led to an increased genuine forgiveness perception, via whole story. However, this indirect effect was only observed for co-reflection in Study 2, albeit marginally significant; it was significant when the victim had indeed indicated forgiveness.

The private, self-directed processes that lead victims to forgive offenders has permeated the philosophies of key forgiveness therapies and interventions (Enright 2001; Worthington 2001). While self-attained forgiveness may be good for victims, this research may suggest that forgiveness interventions could be more effective at facilitating *relationship* repair if there were a joint process in reaching forgiveness because of how offenders perceive the resultant forgiveness. Indeed, co-reflection appears to be more likely than individual reflection to afford offenders a combination of the desirable appraisals that are associated with genuine forgiveness perceptions and with lower unforgiveness perceptions. Towards promoting relationship repair, a forgiveness that develops out of co-reflection may therefore communicate a more positive message to the offender that the victim (and their relationship) is moving on from the hurt caused by the offence, which may help offenders to further reconcile their actions and invest in the relationship (Strelan 2018).

Of course, promoting co-reflection is only useful in cases where the victim desires reconciliation with offender and it is safe for them to do so. Ultimately, it is the prerogative of victims to decide whether they engage with the offenders on the issue and to forgive. Our findings do not place any onus on victims to engage with offenders, to consider offenders’ perspectives, or to communicate to offenders that they are on the same page. Yet, our findings are useful for victims who do wish to talk with the offender, and for victims who do wish to repair or maintain their relationship, because a shared process of working through the offense will lead offenders to view any resultant forgiveness as more genuine.

An alternative reading of the present findings could be that victims’ willingness for engagement *per se* indicates genuine forgiveness and/or less pseudo-forgiveness. That is, offenders may infer that victims feel genuinely forgiving because they were willing to talk about the wrongdoing rather than as a consequence of their co-reflection. It is an empirical question as to whether offenders make key appraisals such as meta-perceived value consensus from victims’ willingness to talk *per se*, or whether they require that offenders perceive that victims have engaged with the incident in some capacity (either via individual reflection or co-reflection). Future research could include an additional condition where the victim indicates a willingness to talk, the victim and offender ostensibly do not get the opportunity to converse, but the victim still expresses forgiveness.

A related future direction is to investigate what elements of co-reflection contribute to genuine forgiveness attributions and its key appraisals. Co-reflection implies that the victim is willing to engage with the offender and work through the transgression. It is possible that offenders could perceive genuine forgiveness if the victim engages with them irrespective of whether they actually work through the transgression, but there could also be unique or additive effects of working through it. Future research could investigate this question by contrasting a co-reflection condition focused on working through a transgression to a victim-offender interaction that follows a transgression but does not focus on resolving the transgression.

### The Key Appraisals That Shape the Meaning of Forgiveness

The present findings shed light on the key appraisals that shape the meaning of victims’ expressed forgiveness. One key appraisal is whether the offender believes that the victim believes in a shared value consensus with them (i.e., meta-perceived value consensus) that is related to offenders making more favourable forgiveness attributions. This finding extends our knowledge of the important, but underexplored, role that meta-perceptions play in moral repair processes.

Another key appraisal is whether the offender perceives the victim to have taken a holistic assessment of the wrongdoing, integrating both perspectives of the wrongdoing (i.e., whole story). The downstream positive forgiveness attributions afforded by whole story also fits with other research that shows there are positive appraisals that come from perceiving that the other person can take one’s point of view during interpersonal conflict. For example, *perceived understanding*, which affords positive relationship attributions (Gordon & Chen 2016), and *perceived perspective-taking*, which affords a sense that the perspective-taker feels empathy towards the person whose perspective has been taken (Berndsen et al. 2018).

However, there were inconsistent findings for processing. In Study 1, processing was negatively related to perceived avoidance and minimisation, and it thus mediated negative indirect effects of co-reflection and individual reflection on avoidance and minimisation. In contrast, in Study 2, processing was positively related to avoidance if the victim had indicated forgiveness, and its negative relationship with (and mediation of negative indirect effects on) minimisation were not replicated. It is possible that these differences emerged due to the differences in methodology between the two studies. Study 1 presented descriptions of victims’ post-transgression engagement that concluded with the expression of forgiveness. This may have suggested that victims had completed their processing of the transgression and that processing resulted in forgiveness. Conversely, offenders in Study 2 recalled real transgressions and post-transgression engagement. Victims’ processing may be a ‘black box’ to offenders after real transgressions because offenders may not be sure whether victims have fully processed the incident (completed) or are continuing to process the incident (ongoing). Our measure of processing captured offenders’ appraisals of whether victims had put effort into processing the incident, but not whether that processing was completed or ongoing. It is possible that if victims express forgiveness but offenders appraise their processing as ongoing, then offenders may see this as victims trying to cut short their processing of the incident (i.e., avoidance). Future research should investigate whether offenders may appraise processing differently depending on whether they perceive victims’ processing to be completed or ongoing.

A cautionary note is that the associations between the key appraisals and the meanings of forgiveness are correlational, so we cannot infer causality for the mediator-outcome relations. For example, future research could manipulate whether the victim indicates that they are processing the incident or considering all sides of the incident (i.e., whole story). Alternatively, future research could use longitudinal designs to test prospective relationships as indications of causal directionality.

Future research could also investigate the potential influence of other contextual variables on offenders’ appraisals. Some possibilities include whether offenders have apologised and/or the perceived severity/intentionality of the wrongdoing which could influence how offenders view engaging in co-reflection and/or victims’ individual reflection on the issue. For example, co-reflection and individual reflection could have counter-acting effects or carry different meaning if offenders feel they have already sufficiently apologised or do not see the wrongdoing as having been intentional or serious enough to warrant consideration (see Adams et al. 2015).

## Conclusion

The act of forgiveness is an important element of moral repair, but forgiveness can mean different things, and so offenders must appraise the meaning of forgiveness (Gollwitzer & Okimoto 2021). The present research demonstrates that offenders interpret victims’ forgiveness based on key appraisals that result from the engagement that follows in the aftermath of wrongdoing. In particular, co-reflection – a joint interactive process of dealing with the wrongdoing – facilitates offenders seeing victims’ forgiveness as genuine because it leads offenders to believe that victims believe in a shared consensus about the violated values and that victims have considered both parties’ perspectives and feelings about the incident. In cases where both parties wish to reconcile, these findings highlight the importance of joint social processes for effective repairing of relationships because offenders are more likely to see victims’ forgiveness as genuine.

## Footnotes

We included three items to assess participants’ perceptions of the seriousness, intentionality, and forgivability of the wrongdoing. The scenario that involved kissing someone who was not your partner was rated as the most serious, most intentional, and least forgivable.

We conducted an analysis that included the engagement factor with scenario as a second factor. Across all DVs there were only two significant moderations (whole story, *p* = .03, and meta-perceived value consensus, *p* = .04), but these were no longer statistically significant after applying an alpha correction for multiple tests. Thus, the results indicate that the effects of engagement hold irrespective of scenario.

Offenders in the co-reflection condition (vs. no reflection) reported greater willingness to reconcile suggesting there may also be downstream behavioural consequences depending on the co-reflection that precedes victims’ forgiveness.

In Study 2, we did not pre-register a replication hypothesis for the indirect effect of co-reflection on unforgiveness via meta-perceived value consensus because of an oversight. However, we still report the tests for this previously pre-registered prediction.
